# The impact of the AstraZeneca suspensions on the public’s vaccination intentions and trust

**DOI:** 10.1093/eurpub/ckac131.040

**Published:** 2022-10-25

**Authors:** M de Vries, L Claassen, M Lambooij, A Timen

**Affiliations:** 1 Infectious Disease Control, RIVM, Bilthoven, Netherlands; 2 Environmental Safety and Security, RIVM, Bilthoven, Netherlands; 3 Nutrition, Prevention and Health Services, RIVM, Bilthoven, Netherlands; 4 Athena Institute, VU University, Amsterdam, Netherlands

## Abstract

**Background:**

In spring 2021, several countries, among which the Netherlands, suspended vaccinations against COVID-19 with the Vaxzevria vaccine from AstraZeneca (AZ) after reports of rare but severe adverse events (SAE). We investigated the impact of this news and the suspension on the Dutch public’s COVID-19 vaccination intentions, COVID-19 vaccination perceptions (attitudes and feelings) and their trust in the government’s COVID-19 vaccination campaign.

**Methods:**

We conducted two surveys (N = 2628), one shortly before the AZ suspension in the Netherlands and one shortly thereafter when all vaccinations were resumed. Chi2 tests were conducted to study changes in COVID-19 vaccination perceptions, intentions and trust before and after the suspension, and differences between perceptions and intentions regarding AZ vaccines compared to COVID-19 vaccines in general. All variables were measured on a 5-point Likert scale.

**Results:**

No significant changes were observed in COVID-19 vaccination perceptions and intentions, but trust in the campaign declined slightly (mean diff.(ΔM)=-0.2, 95% CI=-0.3/-0.2). In addition, compared to COVID-19 vaccinations in general, respondents were less likely to vaccinate with AZ (ΔM=-0.7, 95% CI=-0.7/-0.7), reported less positive vaccine attitudes (ΔM=-0.7, 95% CI=-0.7/-0.7), and more negative feelings (ΔM=0.5, 95% CI = 0.4/0.5).

**Conclusions:**

The news on SAE and the AZ suspension might have caused a decline in trust in the government’s COVID-19 vaccination campaign, as well as negatively impacted AZ vaccination perceptions and intentions. These results stress the need to adapt vaccination policies to anticipated public perceptions and responses following a vaccine safety scare, as well as the importance of informing citizens about the possibility of very rare SAE prior to the introduction of novel vaccines.

**Key messages:**

• Trust in the COVID-19 vaccination campaign declined following the news on rare but severe adverse events (SAE) and the suspension of AstraZeneca vaccines.

• While the news on SAE and the vaccination suspension did not seem to impact COVID-19 vaccination intentions in general, intentions to vaccinate with AstraZeneca were considerably lower.

